# Exploring mitogenome evolution in Branchiopoda (Crustacea) lineages reveals gene order rearrangements in Cladocera

**DOI:** 10.1038/s41598-022-08873-y

**Published:** 2022-03-23

**Authors:** Filippo Castellucci, Andrea Luchetti, Barbara Mantovani

**Affiliations:** 1 Department of Biological, Geological and Environmental Sciences—University of Bologna, via Selmi 3, 40126 Bologna, Italy; 2 Zoology Section, Natural History Museum of Denmark—University of Copenhagen, Universitetsparken 15, 2100 Copenhagen, Denmark

**Keywords:** Evolution, Phylogenetics, Genomics, Genome evolution, Zoology

## Abstract

The class Branchiopoda, whose origin dates back to Cambrian, includes ~ 1200 species which mainly occupy freshwater habitats. The phylogeny and systematics of the class have been debated for long time, until recent phylogenomic analyses allowed to better clarify the relationships among major clades. Based on these data, the clade Anostraca (fairy and brine shrimps) is sister to all other branchiopods, and the Notostraca (tadpole shrimps) results as sister group to Diplostraca, which includes Laevicaudata + Spinicaudata (clam shrimps) and Cladoceromorpha (water fleas + Cyclestherida). In the present analysis, thanks to an increased taxon sampling, a complex picture emerges. Most of the analyzed mitogenomes show the Pancrustacea gene order while in several other taxa they are found rearranged. These rearrangements, though, occur unevenly among taxa, most of them being found in Cladocera, and their taxonomic distribution does not agree with the phylogeny. Our data also seems to suggest the possibility of potentially homoplastic, alternative gene order within Daphniidae.

## Introduction

Species belonging to the class Branchiopoda are distributed worldwide and mainly occupy freshwater habitats, including lakes and ephemeral or temporary ponds, with a few species inhabiting marine environments^1^. The origin of branchiopods is estimated dating back to the Cambrian^2,3^ and the class is currently recognized to include at least 1200 aquatic species (including brine shrimps, tadpole shrimps, fairy shrimps, water fleas)^4^. Branchiopods comprise four extinct and nine extant orders. The superorder Sarsostraca is formed by the extinct order Lipostraca and the extant Anostraca^5^, the latter including around 300 species distributed into the two suborders Anostracina (fairy shrimps) and Artemiina (brine shrimps)^6^. The superorder Calmanostraca comprises the extinct order Kazacharthra and the extant Notostraca, the well-known tadpole shrimps, often considered living fossils due to their morphological stasis. The superorder Cladocera (water fleas) is the most diverse taxon within Branchiopoda and includes the extinct orders Cryptopoda and Proanomopoda, and the extant orders Anomopoda, Ctenopoda, Haplopoda and Onychopoda^5,7^. The order Anomopoda, in particular, includes the genus *Daphnia* O. F. Müller, 1785 whose members are well-known model organisms in ecological and evolutionary studies^8^. The three clam shrimp orders Laevicaudata (which only includes the family Lynceidae), Spinicaudata and Cyclestherida were once included in the group Conchostraca, recently proved to be paraphyletic^5,9^. Cyclestherida and the super order Cladocera are sister clades, forming the taxon Cladoceromorpha which, together with Spinicaudata, belongs to the taxon Onychocaudata^10^. The order Laevicaudata groups with Onychocaudata in the clade Diplostraca. Finally, Notostraca and Diplostraca form the group Phyllopoda, with Anostraca as sister clade^11,12^.

Mitochondrial DNA contains popular molecular genetic markers, widely used for both phylogenetic (mainly for relatively recent divergences) and population genetics studies especially because of quite conserved gene compositions, obvious gene orthology and relative ease of sequencing^13^. The metazoan mitochondrial DNA molecule is generally considered a quite stable part of the genome, about 16 Kb in size and harbouring 37 genes: 13 protein coding genes (PCGs) which encode subunits of oxidative phosphorylation enzymes, two rRNA genes, 22 tRNA genes, and a non-coding control region^14^. However, there are many exceptions to this paradigm, mainly brought to the attention since the advent of massive mitogenome sequencing in the last decades^15^. The most interesting feature of mitogenomes is, probably, the gene order (GO) rearrangement, where the sequential order of genes along the molecule can change, also switching to the opposite DNA strand, and can be subject to gene duplications/deletions^13,14^. An interesting use of GO rearrangement is to help reconstructing the phylogenetic history of organisms, exploiting its relatively rare occurrence and the unlikely event of parallel evolution of identical GOs^16,17^. For example, the close evolutionary relationship between crustaceans and insects, which are in fact both included in the Pancrustacea clade^18,19^, has been further confirmed by the sharing of the same mitogenomic GO (the PanGO pattern), which differs from the so-called ancestral arthropod GO (AAGO pattern) by a single tRNA displacement^20,21^. Given the many examples of GO rearrangements in pancrustaceans at different taxonomic levels^22–31^, the analysis of GO synapomorphy, which may be particularly helpful in phylogenetic reconstruction, may be difficult^25^. In addition to this, several examples of GO parallel evolution have been reported in hexapods^32^.

The first branchiopod mitogenomic study analyzed 24 sequences from 20 species of three anostracan families (Artemiidae, Streptocephalidae and Thamnocephalidae), of the notostracan family Triopsidae, of the spinicaudatan family Limnadiidae and of two cladoceran families, Sididae (Ctenopoda) and Daphniidae (Anomopoda)^33^. This study, on one hand, confirmed the phylogenetic informativeness of the sequence but, on the other hand, it also highlighted significant differences in mitogenome nucleotide composition and substitution rates among the analyzed clades. However, only a small number of GO rearrangements were found, likely due to the limited taxonomic sampling. Since the first study of GO rearrangements in Branchiopoda, though, new mitogenomes have become available in public databases for several lineages, in particular for Cladocera. Along with the previously analyzed dataset, we further considered five new mitogenomes, de novo assembled from genome sequencing data^34,35^, and 37 mitogenomes which become available from NCBI GenBank (39 sequences from recently published papers listed in Suppl. Table S1 and three from the DNAmark Project [https://dnamark.ku.dk/]). Therefore, with respect to the previous analysis^33^, additional 39 species are presently considered, belonging to the following families: Chirocephalidae (Anostraca), Triopsidae (Notostraca), Leptestheriidae (Spinicaudata), Polyphemidae (Onychopoda), Bosminidae, Moinidae and Daphniidae (Anomopoda). With this dataset we highlight a more dynamic nature of branchiopod mitogenome GOs, with some Cladocera showing more structural instability. Moreover, we found identical GOs shared by a few species scattered among different genera within the Daphniidae family.

## Results

### Newly sequenced mitogenomes assembly and annotation

We obtained new mitogenomes from ongoing genome projects of four species^35^: *Lepidurus couesii* Packard, 1875, *Lepidurus apus apus* (Linnaeus, 1758), *Triops longicaudatus* (LeConte, 1846) and *Leptestheria dahalacensis* (Rüppel, 1837). The length of mitogenomes ranges from 15,105 bp (*L. dahalacensis*) to 15,292 bp (*L. couesii*), and they all show 13 PCGs, 22 tRNAs and two rRNAs genes. The *T. longicaudatus* mitogenome has already been characterized in other five samples^36^ and the one presently obtained does not show any peculiar structural variation. The assembly of *Eulimnadia texana* (Packard, 1871) mitogenome resulted in a 14,884 bp long molecule, whose annotation evidenced the same gene content as the newly sequenced ones.

Annotation of mitogenomes downloaded from GenBank was confirmed for all entries, except for the *trnM* duplications in *Diaphanosoma celebensis* Stingelin, 1900 and *Diaphanosoma excisum* G.O. Sars, 1885, and the omission of unassigned regions (URs).

### Dataset construction and phylogenetic analyses

In addition to the five newly assembled mitogenomes, already assembled mitogenomes were further extracted from both genome-wide studies (such as, for example^37,38^) or from single mitogenome sequencing (see Suppl. Table S1 for a full list).

**Figure 1:**
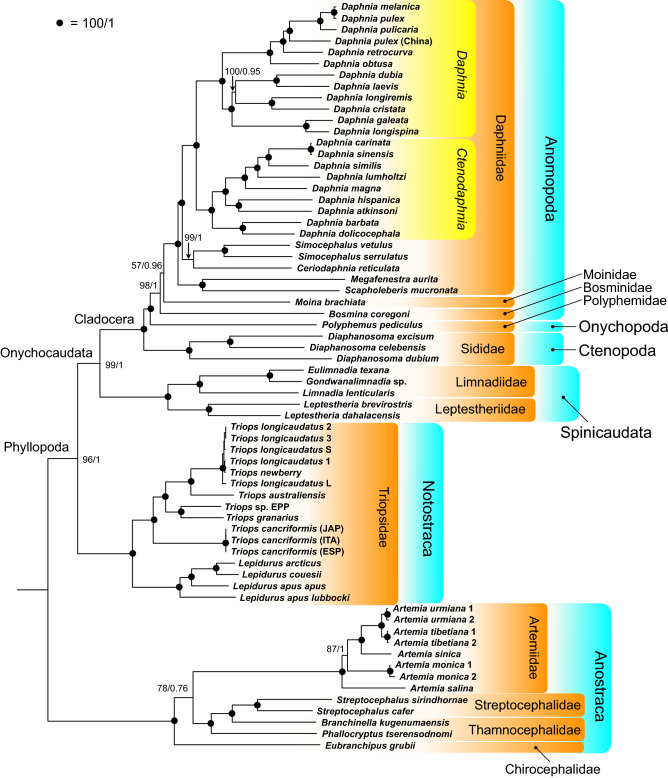
Maximum likelihood phylogenetic tree (−*lnL* = 446,363.518) built on the nucleotide alignment of the 13 mitochondrial protein-coding genes (including 3rd codon positions) + the two rRNA genes using IQTREE. Numbers at nodes represent bootstrap proportion/bayesian posterior probability; full circles indicate maximum nodal support as per upper left legend.

Phylogenetic reconstructions based on the available 66 mitogenomes were performed using three datasets: nucleotide alignments of the thirteen concatenated mitochondrial PCGs, both including and excluding 3rd codon positions to account for possible substitution saturation, plus the two ribosomal RNA genes, and on the aligned PCGs’ amino acids. The three maximum likelihood trees built on the three datasets generally agree with each other, except for a few minor differences (Fig. 1; Supplementary Figure S1). In all phylogenetic analyses the deepest split separates the Anostraca and Phyllopoda clades, which are both recovered with maximum support in the Bayesian inference and high support in maximum likelihood trees with bootstrap values ranging from 96 to 100 (Fig. 1; Supplementary Figure S1).

Within the Anostraca clade, the family Chirocephalidae, here represented by the species *Eubranchipus grubii* (Dybowski, 1860)*,* is a sister group to all the other Anostraca when the nucleotide datasets are considered. The topology changes when analyzing the amino acids dataset, as *E. grubii* results as a sister group to presently considered Thamnocephalidae and Streptocephalidae taxa only (Supplementary Figure S1). On the other hand, the paraphyly of analyzed Thamnocephalidae remains well supported in all analyses (Fig. 1; Supplementary Figure S1). Samples of the *Artemia* genus (family Artemidae) are recovered in a monophyletic clade with maximum nodal support, although the relationship between *Artemia monica* (Verrill, 1869) (formerly indicated as *Artemia franciscana* Kellogg, 1906 but recently revised^39^) and the clade including *Artemia sinica* (Cai, 1989), *Artemia urmiana* Günter, 1899 and *Artemia tibetiana* Abatzopoulos et al., 1998 shows lower support in the nucleotide’s maximum likelihood analyses. Moreover, *Artemia salina* (Linnaeus, 1758) results in sister relationship with all other *Artemia* species in the nucleotide dataset including PCGs 3rd codon positions and in the amino acid dataset, but it is recovered as sister to *A. monica* when 3rd codon positiona are not considered (Fig. 1; Supplementary Figure S1).

Within Phyllopoda, Notostraca is supported with maximum values in all analyses while Onychocaudata is fully supported in the Bayesian inference and highly supported in maximum likelihood trees (bootstrap = 98–99) (Fig. 1; Supplementary Figure S1). The tree topologies within Notostraca are fully overlapping and all main nodes receive maximum support; samples of the genera *Triops* Schrank, 1803 and *Lepidurus* Leach, 1819 are correctly separated in genus-specific monophyletic clades. *Triops cancriformis* (Bosc, 1801) results as sister to all other *Triops* species, and the clade formed by *Triops granariu*s (Lucas, 1864) and *Triops* sp. EPP is sister to the clade *T. longicaudatus* + *Triops australiensi*s (Spencer and Hall, 1895) (Fig. 1; Supplementary Figure S1). In the *Lepidurus* clade, *L. couesii* and *Lepidurus arcticus* (Pallas, 1793) result the most similar to each other, while *Lepidurus apus lubbocki* (Brauer, 1873) is sister to all other *Lepidurus* taxa (Fig. 1; Supplementary Figure S1).

Within Onychocaudata, samples of Cladocera and Spinicaudata are grouped in monophyletic clades with maximum support. In the latter clade, members of Limnadiidae and Leptestheriidae are correctly separated in two monophyletic clusters (Fig. 1; Supplementary Figure S1). Within Cladocera, the three *Diaphanosoma* Fischer, 1850 species (family Sididae) are grouped together and is sister relationship with the other Cladocera considered. Taxa belonging to Daphniidae, the most represented family in our dataset, form a monophyletic clade, with maximum nodal support in all analyses. Its sister relationship with *Moina brachiata* (Jurine, 1820) (family Moinidae) is well supported only in the Bayesian inference, being poorly supported in the maximum likelihood analyses on full nucleotide and amino acid dataset (bootstrap = 57–74), and lacking support when 3rd codon positions are not considered (Fig. 1; Supplementary Figure S1). The genus *Daphnia* and the two subgenera included in the analyses (*Daphnia* and *Ctenodaphnia*) are clearly supported. Phyletic relationships within the subgenera are maintained throughout datasets and analyses, except for two differences. In the subgenus *Daphnia*, the relative placement of *Daphnia obtusa* (Kurz, 1874) and *Daphnia retrocurva* Forbes, 1882 is switched in the amino acid data set, and the relative placement of the *Daphnia cristata* (G.O. Sars, 1862) + *Daphnia longispina* (O.F. Müller, 1776) clade and the *Daphnia galeata* G.O. Sars, 1864 + *D. longispina* clade is switched when PCGs 3rd codon positions are not considered (Fig. 1; Supplementary Figure S1).

### Mitogenomes annotations, gene order and rearrangements

All analyzed mitogenomes include the PCGs and rRNA genes described in the putative ancestral arthropod mitogenome, although the tRNA content could vary to some extent. There are, however, four mitogenomes obtained from the NCBI database which are not complete, namely *Daphnia melanica* Hebert, 1995, *Da. obtusa*, *Scapholeberis mucronata* (O.F. Müller, 1776) and *Polyphemus pediculus* (Linnaeus, 1761).

**Figure 2:**
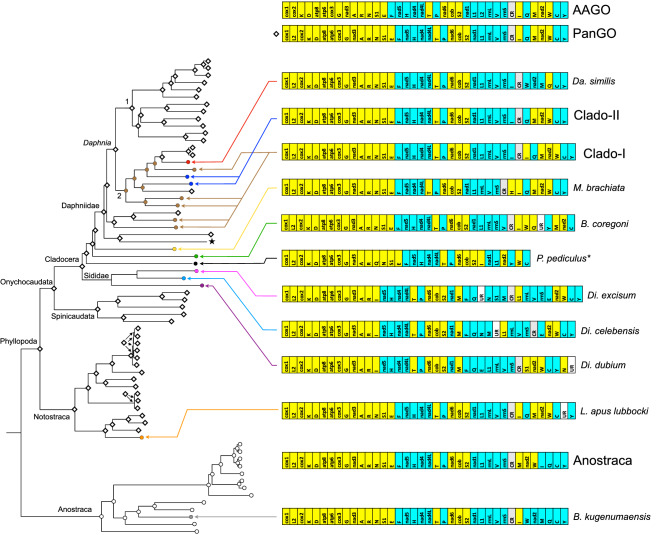
Schematic drawing of observed mitogenome gene orders (GOs) with their phylogenetic distribution. Numbers in the *Daphnia* clade indicate the two subgenera *Daphnia* (1) and *Ctenodaphnia* (2). Yellow and cyan genes colour indicate they are coded on the two different strands. *AAGO* Ancestral Arthropod Gene Order, *PanGO* Pancrustacea Gene Order. Clado-I gene order pattern is shared by: *Da. lumholtzi*, *Da. barbata*, *Da. dolichocephala*, *C. reticulata* and *Si. serrulatus*. Clado-II gene order pattern is shared by: *Da. magna* and *Da. hispanica*. Asterisk on *P. pediculus* indicates it is an incomplete genome. The star indicates the position of *Sc. mucronata* whose GO could be either Clado-I or Clado-II, but due to mitogenome incompleteness it cannot be determined. Diamonds at nodes indicate ancestral GOs identical to the PanGO pattern, while circles indicate different GOs (brown = Clado-I pattern; white = Anostraca pattern), as reconstructed with MLGO method.

Among the analyzed 66 branchiopod mitogenomes we found evidence of 13 distinct GOs (Fig. 2). The most common one characterizes the majority of Phyllopoda taxa: all Spinicaudata, all Notostraca, but *L. apus lubbocki* showing an unassigned region (UR)^33^, and 17 out 32 Cladocera. This pattern is identical to the PanGO pattern, which differs from the AAGO one because of the reverse transposition of the *trnL2* gene^20,21^.

The other 15 cladoceran taxa showed nine different GOs, three being found in the family Daphniidae. In the genus *Daphnia*, the PanGO pattern can be found in all species belonging to the subgenus *Daphnia,* as well as in three species of the subgenus *Ctenodaphnia*. The remaining *Ctenodaphnia* taxa exhibited three distinct GOs. The most common one, hence referred to as Clado-I, is shared between *Daphnia lumholtzi* G.O. Sars, 1885, *Daphnia barbata* Weltner, 1898 and *Daphnia dolichocephala* G.O. Sars, 1895, and it was also found in the other daphniids *Ceriodaphnia reticulata* (Jurine, 1820) and *Simocephalus serrulatus* (Koch, 1841). This GO differs from the PanGO pattern because of the duplication and reverse transposition of the *trnI* gene. Another GO, hence termed Clado-II, characterizes the two species *Daphnia magna* Straus, 1820 and *Daphnia hispanica* Glagolev & Alonso, 1990 and showed a reverse transposition of the *trnI* with respect to the PanGO pattern. The third GO can be observed only in *Daphnia similis* Claus, 1876 and differs from the PanGO pattern by the reverse transposition of the *trnI* and *trnQ*-*trnM*-*nad2*-*trnW* reversal (Fig. 2).

As far as the other cladoceran taxa are concerned, the GOs pattern looks even more complex. Species representative of Moinidae (*M. brachiata*), Bosminidae (*Bosmina coregoni* Baird, 1857) and Polyphemidae (*P. pediculus*) all show different GOs with respect to the PanGO pattern. *M. brachiata* shows the *trnH* reverse transposition; *B. coregoni* presents the *trnW* transposition and the *trnQ* reversal flanked by a 50 bp-long UR; finally, *P. pediculus* exhibits the transposition of *trnI*, *trnQ* and *trnW* (although, being only partially sequenced, further rearrangements cannot be excluded). The incomplete mitogenome of *Sc. mucronata* showed a *trnI* located between the *rrnS* and the CR, as in the Clado-I and Clado-II patterns; however, since it lacks the portion between CR and *nad2* gene, it is not possible to attribute it to one of the two patterns.

In the family Sididae, the three analyzed samples, all belonging to the genus *Diaphanosoma*, showed three different GOs. Because of the high GO diversity and the consequent complexity in inferring the rearrangement pattern, these mitogenomes were compared with each other and with the PanGO pattern in a phylogenetic framework using TreeREx^40^. Unfortunately, data are not clear enough to result in a highly consistent ancestral reconstruction scenario (Supplementary Figure S2). According to the phylogenetic relationships, the two most similar GO are those of *Di. celebensis* and *Di. excisum* which share a duplication of the *trnM* and only differ for (i) the lack of *trnS1* in the former species and (ii) the transposition of the control region in the latter one (Fig. 2; Supplementary Figure S2). The scenario of possible rearrangements between these two GOs and the one of the sister species *Diaphanosoma dubium* Manujlova, 1964 includes several events of transposition, reverse transposition, and reversal (Supplementary Figure S2). Finally, rearrangements leading from the putative ancestral PanGO pattern to that of Sididae are inferred as even more complex, comprising both a transposition and two possible tandem duplication and random loss events (Supplementary Figure S2).

The Anostraca GO pattern is slightly different from the PanGO pattern, showing a transposition of the *trnM*-*nad2*-*trnW* and a reversal of *trnI* (Fig. 2), and characterizes all anostracan taxa but the thamnocephalid *Branchinella kugenumaensis* (Ishikawa, 1895). The latter mitogenome, in fact, shows a reversal rearrangement involving the segment *trnM*-*nad2*-*trnW*-*trnI* (Fig. 2).

The MLGO method reconstructed the PanGO pattern as the ancestral one, except for the Anostraca clade (Fig. 2). Furthermore, the PanGO pattern was reconstructed at all Phyllopoda nodes with only two exceptions. The first one concerns the *Ctenodaphnia* clade, for which Clado-I was indicated as the ancestral pattern (Fig. 2). It is to be noted, though, that the common ancestor of *Daphnia* and *Ctenodaphnia* resulted to have the PanGO pattern (Fig. 2). The second exception concerns internal nodes of the *Diaphanosoma* clade, for which it was inferred a GO identical to the one of *Di. celebensis* but with a *trnS1* gene instead of the duplicated *trnM* gene.

## Discussion

In the present work we report on the analysis of mitogenomes from some Branchiopoda lineages by providing new data and including data from the NCBI public database. Overall, the evolution of 66 mitogenomes has been investigated through maximum likelihood and Bayesian phylogenetic analyses based on PCGs and rRNA genes, and gene order structural analysis. Despite the lack of some important lineages, such as Laevicaudata and Cyclestherida, data presented here point out interesting features of branchiopod mitogenomes.

The phylogenetic relationships among major lineages of branchiopods, after being debated for a long time, have been recently better clarified through phylotranscriptomic and phylogenomic approaches^10,12^. Our analyses of the three datasets (i.e., PCGs with and without 3rd codon positions + rRNAs; PCGs amino acids) are in agreement with each other, the only exception being the possible paraphyly of Anostracinae (here represented by Chirocephalidae, Thamnocephalidae and Streptocephalidae taxa) using the nucleotide dataset, which was also found in the phylotranscriptomics analysis, and are in line with the current accepted phylogeny. Moreover, our phylogenetic analyses are in good agreement with most recent molecular studies. For example, results pointed out in previous studies on Notostraca^41–43^ are hereby confirmed, including the close relationship between the sample *Triops* sp. EPP and *T. granarius*^44^, and the same holds for Spinicaudata^45^. As far as Cladocera is concerned, the phylogenetic relationships between orders are in line with previous analyses^10,12,38,46^, although Haplopoda representatives are missing here. Moreover, our results are consistent with Daphniidae mitogenome phylogenies obtained by Cornetti and colleagues^38^ and with a very recent mitogenomic analyses which was published during the writing of the present work^46^. Though, it is worth noting that nuclear genes phylogenomic analyses pointed out a different clustering pattern of the genera *Megafenestra* Dumont & Pensaert, 1983, *Scapholeberis* and *Simocephalus*, suggesting a possible limitation of mitochondrial genes phylogenetic signal^10,38^.

The most striking feature of our new analysis is the finding of several different GOs and their taxonomic distribution. As previously noticed, the GO in Branchiopoda was considered rather stable, with just few exceptions^33^. In the present analysis, thanks to the increased taxon sampling, even though still limited with respect to the entire branchiopod diversity, a more complex picture emerges. First of all, even if most mitogenomes show the PanGO pattern, we found several rearrangements. In the second place, we found GO rearrangements occurring unevenly among taxa, with Cladocera showing most of them. Finally, the taxonomic distribution of GO rearrangements does not generally agree with the phylogeny.

It has been recently pointed out that published problematic mitogenomes are increasing, with potential bias with respect to species identification, bad annotation, or assembly issues^47^, suggesting caution when reusing literature data. In the present work, rearranged mitogenomes have been taken from literature studies which, however, implemented several methods to avoid potential assembly bias (as in^37,38^) or gave the same GO although being produced in independent studies (like *D. hispanica*^38^ and *D. magna*^48^). Moreover, annotations were further validated in the present study by using the same pipeline for all analyzed mitogenomes and we did not find signs of potential artifact in genome assembly. Therefore, potential biases were reduced as much as possible, although they cannot be entirely excluded.

Mitogenome gene rearrangements are frequently observed in many metazoan taxa^17^. Within Pancrustacea, for example, several different GOs have been recorded at various taxonomic levels. In a recent survey in Hexapoda, one of the most studied groups of metazoans, about 47% of mitogenomes were found rearranged with respect to the PanGO pattern, which is the prevalent GO in the class^32^. In non-branchiopod crustacean taxa, although much less studied, still several variations with respect to the PanGO pattern have been observed: for example, in Decapoda^26,28,29^, Amphipoda^23,31^, Copepoda^24,27^ and Ostracoda^22,30^.

Interestingly, the observed rearrangements were also found unevenly distributed, again in line with data on Hexapoda^32^ and other crustaceans^28^. Reasons behind GO rearrangements are still debated, as there is no general evidence of association with a specific driving force. Yet, either molecular mechanisms^49–52^ or life history and ecological traits^27,28^ have been tentatively proposed, although none of them seem generalizable^13,28,53^. During the writing of the present paper, a large-scale mitogenomic analysis focusing on Cladocera was published which highlighted the same GOs instability in this clade^46^ as we found in the present analysis. At the moment, though, it is difficult to formulate a reliable hypothesis to explain the observed distribution of GO rearrangements on the basis of clade-specific traits.

The decoupling of the phylogenetic pattern and the GOs distribution pattern is somehow surprising, especially considering that Clado-I and Clado-II GO patterns appear, in the present study, as potentially homoplastic^54^. In the analysis of Xu and collaborators^46^ this went unnoticed probably due to the differences in the *Ctenodaphnia* dataset, which is wider in the present analysis as we included the broad taxon sampling of this subgenus from Cornetti et al.^38^. The MLGO analysis indicated the PanGO pattern as ancestral to Cladocera and, more in particular, it was also found ancestral to the whole Daphniidae family, to the whole *Daphnia* genus and to the most recent ancestor of *C. reticulata* and of *Si. serrulatus*. On the other hand, the same analysis indicated the Clado-I pattern as ancestral to the subgenus *Ctenodaphnia*, although this clade includes three taxa exhibiting the PanGO pattern and further three taxa with different GOs (two with the Clado-II one, and *Da*. *similis* with its own GO). The occurrence of the Clado-I pattern in phylogenetically separate lineages, such as *Ctenodaphnia* spp., *C. reticulata* and *Si. serrulatus*, that started to diverge more than 150 million years ago^10^, together with the MLGO inference of PanGO pattern as ancestral to Daphniidae, could be explained as the result of a multiple, independent evolution of the same GO pattern or because of collateral evolution, *i.e.* the differential inheritance of polymorphic characters evolved in an ancestral population or shared after hybridization^55^. Independent evolution of GOs has been found occurring frequently in Hexapoda and in other metazoans, both between closely related and distantly related species^32,56^. Data collected for this study do not allow to test whether collateral evolution may have had a role in determining the observed GOs pattern of distribution. Though, it is interesting to note that presently observed, potentially homoplastic GOs differ from each other and from the PanGO pattern by the duplication and/or the positioning of the *trnI* gene(s). In the insect order Hymenoptera, tRNA genes are known to be subject to rearrangement more than other genes, thus suggesting the selective neutrality of their position along the mitochondrial GO^57^. Therefore, it is possible to hypothesize that the ease with which tRNA gene positions rearrange could make more likely their multiple, independent occurrences. In line with these findings, the MLGO inference of Clado-I pattern as ancestral to the *Ctenodaphnia* clade may have two further possible implications: (i) the Clado-II pattern and the *Da. similis* GO were derived from the Clado-I one, likely through the loss of the *trnI* gene in the ancestral position (*i.e.*, downstream the control region in the PanGO pattern) and a further rearrangement in *Da. similis*; (ii) three *Ctenodaphnia* species, that do not cluster together, actually showed the PanGO pattern, suggesting independent reversals to the ancestral GO possibly obtained after the *trnI* loss at the derived position (*i.e.*, upstream the control region in the PanGO pattern).

The observed intriguing pattern of GO evolution in Cladocera still remains, therefore, to be clarified: although it clearly appears to derive from the ancestral PanGO pattern, its evolution in some parts of the tree cannot be reconstructed because of uncertainty likely linked to missing data. As previously suggested^46^, the analysis of more mitogenomes and widening the taxon sampling in this region of the branchiopods’ tree of life, to include other major lineages and high quality mitogenome assemblies, will allow to better elucidate this issue.

## Materials and methods

### New mitogenome assembly and annotation

Total DNA was extracted from individual specimens of *L. couesii*, *L. apus apus*, *T. longicaudatus* and *L. dahalacensis* using the DNA extraction kit (STRATEC), after dissection for gut removal. Whole genome sequencing has been carried out on Illumina HiSeqX platform (Macrogen Inc., South Korea), paired end (2 × 150 bp) and library insert size = 350 bp. Raw reads were quality trimmed and deprived of adapters using Trimmomatic v. 0.35^58^, with default settings. A random subset of 4 million read pairs per sample was de novo assembled from using SPAdes v. 3.11.1 with default parameters^59^. No reference sequences have been used to guide the assembly. For *Eulimnadia texana* 4 four million random read pairs from genome sequencing raw reads were used^34^ (SRA accession number SRR4787251; mitogenome available in Suppl. Data S1). For each assembly, the contig containing the mitogenome was found through a BLAST search using as query previously sequenced cox1 genes for *L. couesii* (NCBI GenBank acc. no. DQ148287), *L. apus apus* (DQ834543), *T. longicaudatus* (JX110647) and *L. dahalacensis* (DQ872786), and the rrnS gene for *E. texana* (AY779667). Mitogenome annotation was performed de novo using MITOS2^60^, with RefSeq 89 Metazoa as reference parameter. Where necessary, gene boundaries were manually corrected by similarity with published branchiopod mitochondrial genomes^33^. Genes for tRNAs were further confirmed by means of ARWEN v. 1.2.3^61^.

### Dataset construction and phylogenetic analysis

All available branchiopod mitogenomes were obtained from NCBI GenBank (last accessed in April 2021), therefore producing, together with the presently de novo assembled mitogenomes, a dataset of 66 sequences for 57 species (Supplementary Table S1). Two hexapod mitogenomes, belonging to *Thermobia domestica* Packard, 1873 and *Reticulitermes flaviceps* (Oshima, 1908) (Supplementary Table S1), were used as outgroup for phylogenetic analyses. Annotations of mitogenomes from the database were checked with the same pipeline to verify possible mistakes and to better define boundaries of possible Unassigned Regions (UR). The latter were considered only when longer than 50 bp.

All the thirteen protein-coding mitochondrial genes (PCGs) and the two ribosomal RNAs were aligned using MAFFT v. 7.397^62^ using respectively the L-INS-i and E-INS-i algorithms for PCGs and rRNAs, respectively, and single gene alignments were concatenated. Maximum likelihood phylogenetic reconstruction was performed on the IQ-TREE web server^63^ using the automatic evaluation of the partitioning scheme and substitution model (Supplementary Table S2), edge unlinked partition and FreeRate heterogeneity. Ten runs were performed with 1000 Ultrafast bootstrap replicates each and the tree with the best likelihood score was chosen. Bayesian inference for tree reconstruction was performed only on the full nucleotide dataset using MrBayes v3.2.7^64^, with best partition scheme and substitution models determined using PartitionFinder2 v2.1.1^65^ (Supplementary Table S2). Two runs, each including 10,000,000 generations, were performed with sampling every 1000 trees. Convergence was assessed using Tracer v1.7^66^ and by checking PSRF values and average standard deviation of split frequencies.

### Gene order analysis

Gene order (GO) rearrangements were pairwise compared through the analysis of common intervals, *i.e.* group of consecutive genes occurring in multiple genomes, using the software CReX^67^ with default parameters. The software TreeREx^40^ was also used with designers’ suggested settings (available at http://pacosy.informatik.uni-leipzig.de/185-0-TreeREx.html). It performs the same analysis of CReX but in a phylogenetic framework, by means of ancestral GOs reconstruction: this is particularly useful when pairwise GO comparison is difficult because of high rearrangement levels. On the other hand, both CReX and TreeREx do not manage GOs with duplicated or missing genes: since in our branchiopod dataset there are several duplicated or missing genes, especially regarding tRNA genes, gene duplications/deletions were treated manually and TreeREx was limited to the analysis of the family Sididae.

The attempt to reconstruct ancestral GOs was carried out by means of the MLGO (Maximum Likelihood for Gene Order analysis^68^) method, using the presently obtained phylogenetic tree, based on PCGs (including 3rd codon positions) + rRNAs nucleotide alignment, as backbone tree.

## Supplementary Information

Supplementary Information.

Supplementary Figure S1.

Supplementary Figure S2.

Supplementary Tables.

## Data Availability

Newly sequenced and assembled mitogenomes have been deposited in NCBI GenBank under the following accession numbers: *Lepidurus couesii*, OL757477; *Lepidurus apus apus*, OL757476; *Triops longicaudatus*, OL757475; *Leptestheria dahalacensis*, OL757474.
