# Supplementary figures and images for: Exploring mitogenome evolution in Branchiopoda (Crustacea) lineages reveals gene order rearrangements in Cladocera
Source: Sci Rep. 2022 Mar 23;12:4931. doi: 10.1038/s41598-022-08873-y (PMC8942981; doi:10.1038/s41598-022-08873-y)

(a)

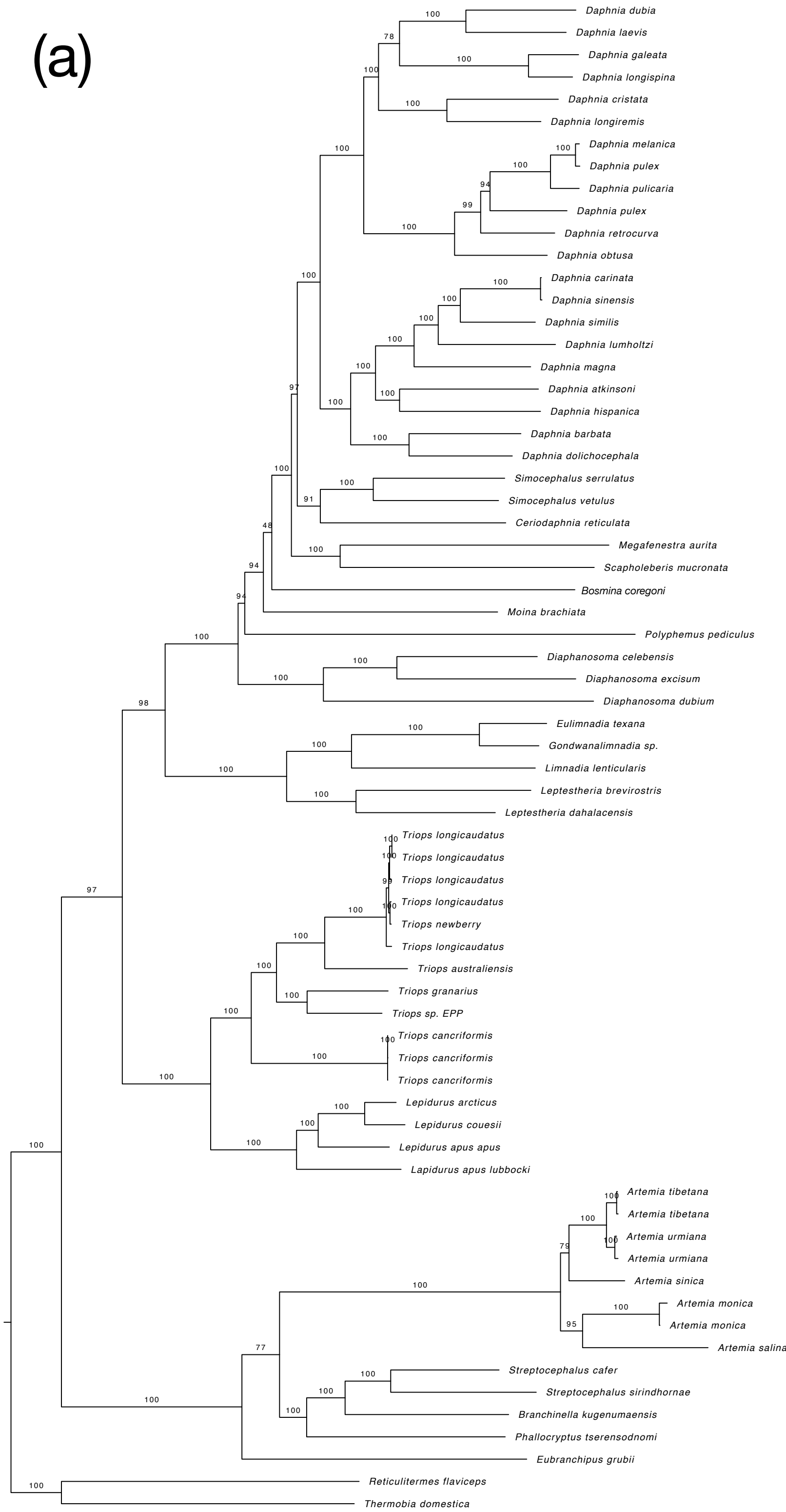

(b)

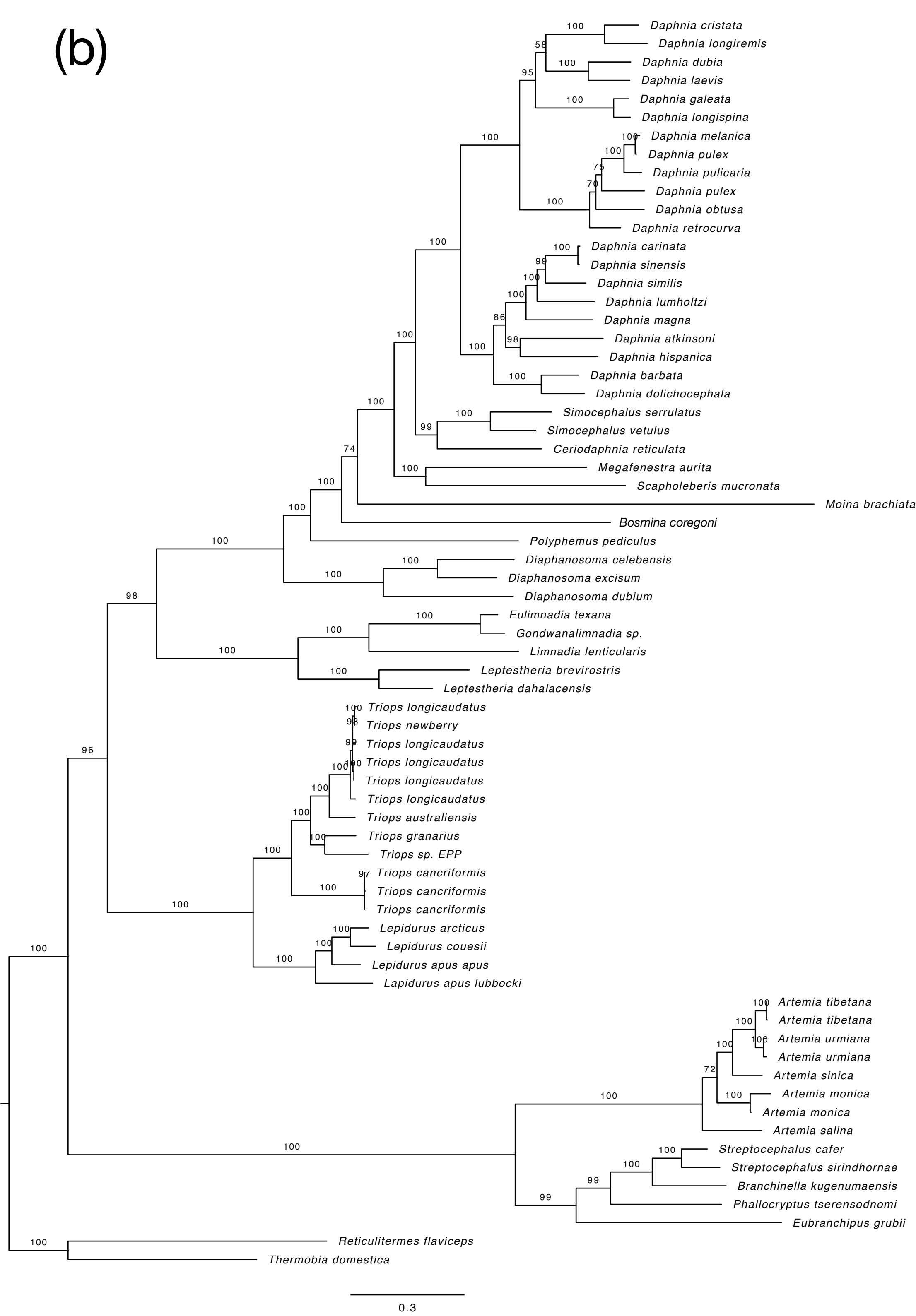

Supplement: Supplementary file 2 — Supplementary Figure S1. [file 41598_2022_8873_MOESM2_ESM.pdf]
