# Supplementary material for: Exploring mitogenome evolution in Branchiopoda (Crustacea) lineages reveals gene order rearrangements in Cladocera
Source: Sci Rep. 2022 Mar 23;12:4931. doi: 10.1038/s41598-022-08873-y (PMC8942981; doi:10.1038/s41598-022-08873-y)
Supplement: Supplementary file 3 — Supplementary Figure S2. [file 41598_2022_8873_MOESM3_ESM.pdf]

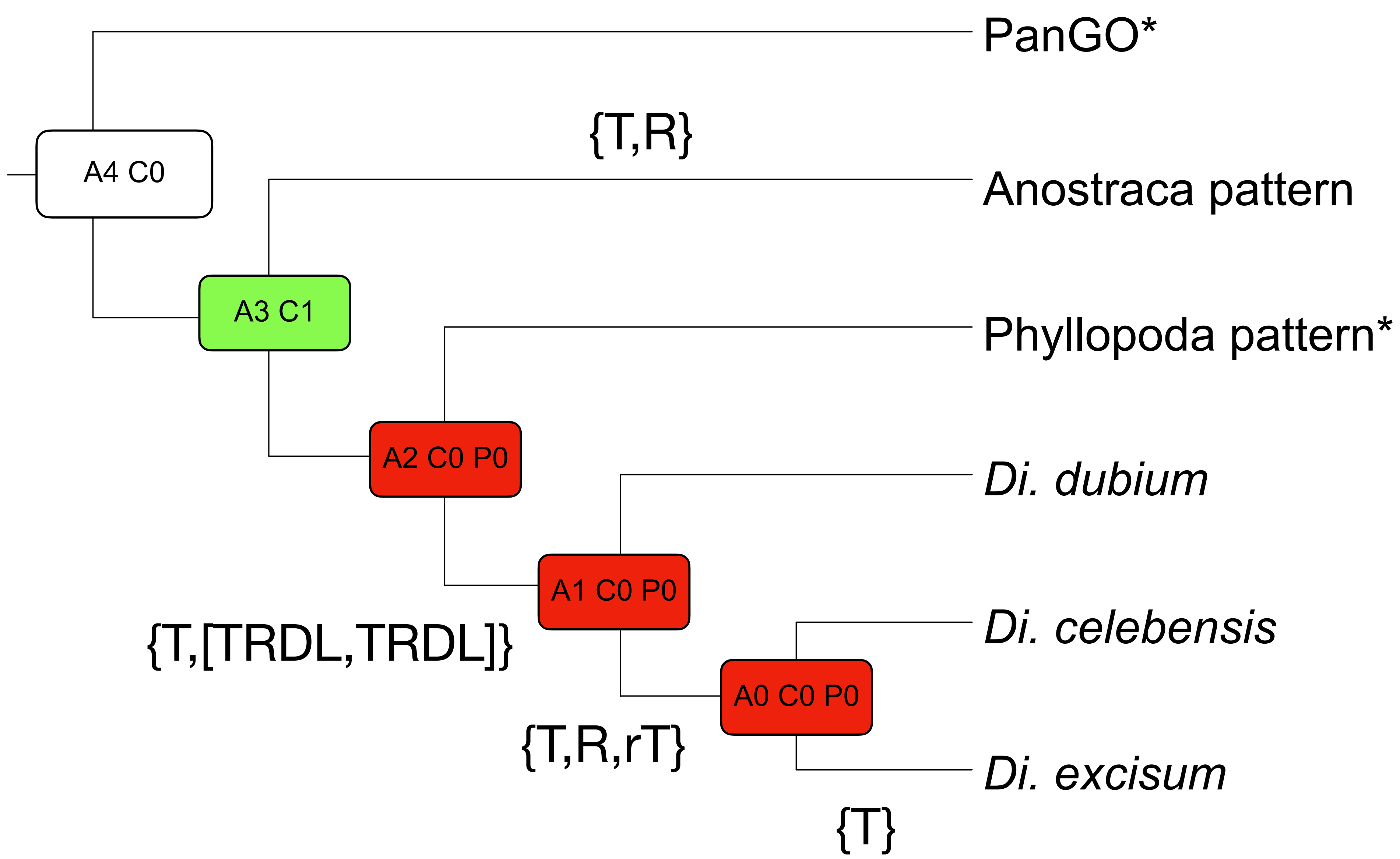

Legends of rearrangemens:

T=transposition
R=reversal
rT=reverse transposition
TRDL=tandem duplication random loss

```
Scenario to Anostraca pattern
unordered{
            transposition(I Q ,M nad2 W ,)
,           reversal(I )
,} complete=1

**************************************************************

Scenario to A1
unordered{
             transposition(N ,E F nad5 H nad4 nad4L T P nad6 cob S2 nad1 L1 rrnL V rrnS CR I Q M nad2 W C Y ,)
,            ordered[
                        reversal(E )
,                       TDRL(F I Q ,E nad5 H nad4 nad4L T P nad6 cob S2 nad1 L1 rrnL V rrnS CR ,)
,                       TDRL(nad5 H nad4 nad4L T P nad6 cob S2 nad1 I M ,E F L1 rrnL V rrnS CR Q ,)
              ] complete=1
,} complete=1

**************************************************************

Scenario to A0
unordered{
             transposition(E ,L1 rrnL V rrnS CR ,)
,            reversal(L1 )
,            reverse transposition(N ,E L1 rrnL V rrnS CR nad2 W C Y ,)
,} complete=1

**************************************************************

Scenario to Di. excisum
             transposition(L1 rrnL V rrnS ,CR ,)

**************************************************************
```

Supplementary Figure S2 - TreeREx analysis output of the comparison between *Diaphanosoma* spp. mitogenome gene order and the other main gene order patterns. Asterisks indicate that PanGO and Phyllopoda pattern are the same gene order.
